# Mechanical stability of the proximal tibia with different bone formations after plate removal in medial opening-wedge high tibial osteotomy: a finite element analysis

**DOI:** 10.1186/s13018-024-05373-9

**Published:** 2024-12-19

**Authors:** Chul-Young Jang, Kyoung-Tak Kang, Hyongtaek Hong, Min Jung, Sungjun Kim, Je-Hyun Yoo, Sung-Hwan Kim

**Affiliations:** 1 Department of Orthopedic Surgery, Yonsei University College of Medicine, Seoul, Republic of Korea; 2 Department of Mechanical Engineering, Yonsei University, 50 Yonsei-ro, Seodaemun-gu, Seoul, Republic of Korea; 3 Skyve R&D Lab, 11, Dangsan‑Ro 41‑Gil, Yeongdeungpo‑Gu, Seoul, Republic of Korea; 4 Department of Orthopedic Surgery, Severance Hospital, Yonsei University College of Medicine, Seoul, Republic of Korea; 5 Arthroscopy and Joint Research Institute, Yonsei University College of Medicine, Seoul, Republic of Korea; 6 Department of Radiology, Gangnam Severance Hospital, Yonsei University College of Medicine, Seoul, Republic of Korea; 7 Department of Orthopedic Surgery, Hallym University Sacred Heart Hospital, Hallym University College of Medicine, Anyang, Republic of Korea; 8 Department of Orthopedic Surgery, Gangnam Severance Hospital, Yonsei University College of Medicine, 20 Eonju-ro 63-gil, Gangnam-gu, Seoul, 06229 Republic of Korea

**Keywords:** High tibial osteotomy, Plate removal, Optimal timing, Finite element analysis

## Abstract

**Background:**

No clear agreement exists on the degree of bone formation required to remove a metal plate without correction loss after medial opening-wedge high tibial osteotomy (MOWHTO). We aimed to investigate the mechanical stability of the proximal tibia with different bone formations after plate removal in MOWHTO using finite element models and determine the extent of bone formation when the plate can be removed without correction loss.

**Methods:**

The MOWHTO models with 5, 10, and 15 mm opening gaps were generated. The mechanical stability of proximal tibial models with different extents of bone formation (from the lateral cortex of the osteotomy wedge to 20% (zone 1), 40% (zone 2), 50% (zone 2.5), 60% (zone 3), 70% (zone 3.5), 80% (zone 4), and 100% bone formation medially) after plate removal was analyzed using finite element analysis. Bone stress and strain and micromotion were evaluated to investigate fracture risk and bone stability, respectively, in various types of tibial models.

**Results:**

Peak von Mises stress was lower than yield strength when bone formation reached zone 3.5 (70%) or more in 5- and 10-mm osteotomy gap models, and zone 4 (80%) or more in a 15-mm gap model. Maximal principal strains were lower than 6,130 microstrain when bone formation reaches zone 3.5 (70%) or more in models with osteotomy gaps of 5, 10, and 15 mm.

**Conclusions:**

This indicates that plate removal without correction loss after MOWHTO may be possible when bone formation reaches zone 3.5 (> 70%) or more during 5- and 10-mm osteotomy gap corrections, and zone 4 (> 80%) or more during 15-mm gap correction. The present study results suggest that it would be safer to perform plate removal after obtaining sufficient bone formation rather than performing it near the osteotomy gap center (50%) to avoid correction loss considering both coronal and sagittal plane aspects.

## Background

Medial opening-wedge high tibial osteotomy (MOWHTO) is a surgical intervention used to treat varus malalignment associated with medial osteoarthritis in middle or early old age [1–3]. This procedure shifts the weight-bearing axis of the lower limb laterally in the coronal plane, increases the width of the medial joint space, and reduces the medial compartment load to postpone transition to total joint surgery [4–9]. Compared with lateral closing-wedge osteotomy, MOWHTO has the advantage of easy adjustment of correction degree and a low risk of peroneal nerve damage [10, 11]. However, lateral hinge fractures, implant breakage, and correction loss occur more frequently after MOWHTO due to the relatively unstable structure generated by the opening gap [1, 12, 13]. Therefore, long and bulky metal plates with locking screws have traditionally been preferred for fixation in MOWHTO. A rigid and bulky metal plate improves stability around the osteotomy site; however, many patients complain of discomfort at the fixation site. Niemeyer et al. [14] reported that after MOWHTO, 40.6% of patients complained of local discomfort and irritation associated with the implant. Darees et al. [15] reported that 41.6% of patients treated with MOWHTO removed the plate because of the discomfort. Most Asian or lean patients with a thin subcutaneous layer visit an outpatient clinic to remove the metal plate before achieving complete bone union because of discomfort.

No clear agreement exists on the degree of bone formation required for plate removal without correction loss after MOWHTO. Staubli et al. [16] reported that approximately 90% of patients gained full consolidation of osteotomy site on simple radiography, computed tomography (CT), and magnetic resonance imaging in 1 year. Brinkman et al. [17] did not suggest hardware removal 1.5 years after corrective osteotomy. However, Kobayashi et al. [18] reported that the extent of posterior cortex union that reached zone 3 (40–60% of healing) was sufficient for metal plate removal. Goshima et al. [19] suggested that hardware removal was possible without correction loss after MOWHTO when posterior cortex union reached the midpoint (> 50%) of osteotomy space. However, studies to determine the point where correction loss occurs after plate removal in MOWHTO with various degrees of correction are still insufficient, and finding this point using only clinical studies is challenging. Furthermore, to the best of our knowledge, no study has evaluated the mechanical stability of proximal tibial models with metal plates removed after MOWHTO.

We aimed to investigate the mechanical stability of the proximal tibia with different bone formations after plate removal in MOWHTO using finite element (FE) models, and to determine the degree of bone formation where the plate can be removed without correction loss. The present study hypothesized that a possible point for plate removal without the risk of correction loss would exist in the < 50% progression zone of bone formation on anterior-posterior (AP) radiographs.

## Methods

### Development of proximal tibia model with plate removal after MOWHTO

This study was approved by the institutional review board of Yonsei University Gangnam Severance Hospital (IRB No. 3-2024-0263). This study was conducted in accordance with the Declaration of Helsinki, and informed consent was obtained from the patient participating the present study. Cross-sectional images of the lower extremities of a 62-year-old Asian female were obtained using 64-channel computed tomography (CT) with a slice interval of 0.1 mm. Three-dimensional geometric contour of the tibia was created by stacking the CT cross-sectional images using Mimics (version 17.021.0; Materialize Inc., Belgium). Bone surfaces were modified into more sophisticated solid models using computer-aided design (CAD) software and Unigraphics NX (version 2021; Siemens PLM Software, Torrance, CA). An FE mesh was generated using HyperMesh (version 8.0; Altair Engineering).

**Fig. 1:**
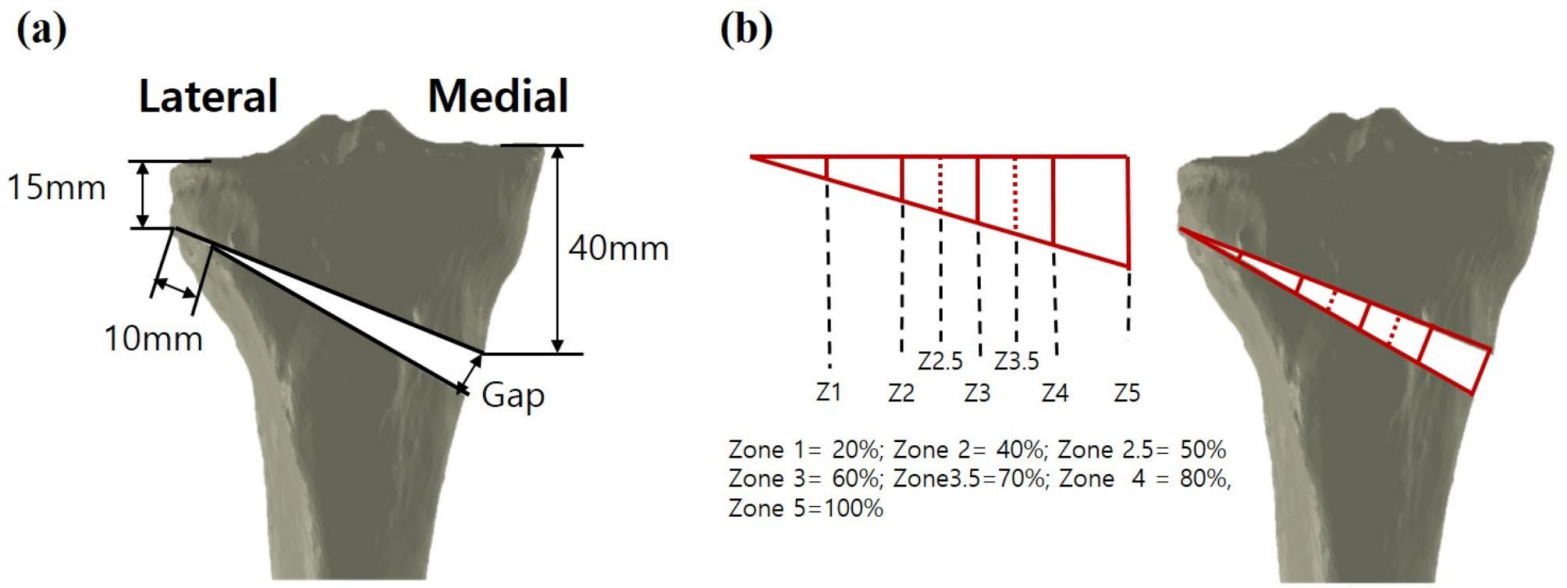
Specifications of the MOWHTO used in this study (**a**) and osteotomy filling index with seven identified zones (**b**)

The constructed healthy tibia model was used to simulate MOWHTO with rotation of the distal part of the tibia in the coronal plane to create a valgus correction angle. The MOWHTO models with 5, 10, and 15 mm gaps were generated by removing a single-plane wedge-shaped osteotomy bone at the proximal tibia while leaving a 10 mm hinge from the lateral cortex (Fig. 1a) [20].

A commercially used fixation plate, TomoFix (DePuy Synthes, Warsaw, IN, USA), was considered as the worst case in terms of bulkiness, used in MOWHTO simulation, and modeled three-dimensionally using the CAD program and Unigraphics NX (version 7.0; Siemens PLM Software, Torrance, CA, USA). The plate model was virtually implanted into the tibia to simulate MOWHTO fixation and then removed to create a proximal tibia model with bony deficiency at the opening wedge and screw holes. The plate had a length of 112 mm with 4 proximal screws and 4 distal screws, 5 mm screw diameter for proximal and distal, 65 mm proximal screw length, and 36 mm distal screw length [21] .

To create proximal tibial models with different degrees of bone formation at the time of hardware removal, we further subdivided the osteotomy filling index proposed by Brosset et al. [22]. The tibia models with different union progressions at the time of plate removal were generated by artificially forming cortical bone with trabecular bone inside formation from the lateral cortex of the osteotomy wedge to 20% (zone 1), 40% (zone 2), 50% (zone 2.5), 60% (zone 3), 70% (zone 3.5), 80% (zone 4), and 100% medially (Fig. 1b).

As for the tibia, cortical bone was considered linear elastic, isotropic, and homogeneous material with an assumed Young’s modulus (E), E = 17,000 MPa and Poisson ratio (ν), ν = 0.33 [23, 24]. Cancellous bone was simulated as a linear isotropic material with E = 910 MPa and v = 0.2 [25]. A “tie” contact condition was applied assuming full constraints between bone and bone. The distal end of the tibial bone was assumed to be fully constrained in all tests [26, 27].

Mesh convergence of the FE model was investigated to complete the modeling. Mesh convergence was defined as the maximum displacement of trabecular bone, which was within 95% of the pressure of the next two smaller mesh sizes [28]. The average FE size was 0.8 mm for the cortical and cancellous bone. To avoid singularities and achieve convergence in the FE model bone region, a graded bone model was implemented. A mesh size of 0.6 mm on at the posterolateral end of the wedge region, and 0.8 mm for other regions. Quadratic tetrahedral elements of type C3D10 was applied. The numbers of created finite elements were as follows: cortical bone, 77,576 and cancellous bone, 178,726.

### Loading and boundary conditions

**Fig. 2:**
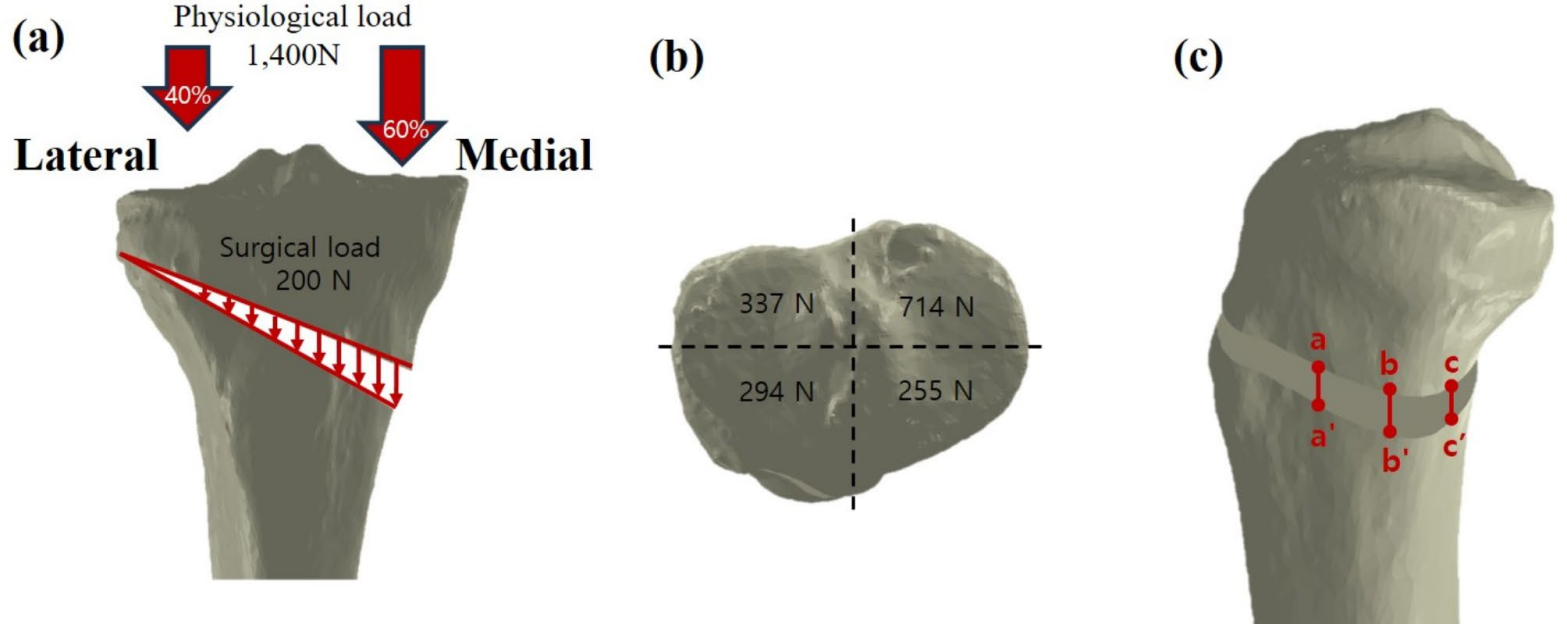
Loading conditions used in the present study; physiological and surgical loads (**a**), and loads on the four regions of the tibial plateau (**b**). Three edges aa’, bb’, and cc’ along the medial cortex were defined to evaluate the height changes in loading condition (**c**)

Physiological loading and intervention-induced compression were applied to the MOWHTO models. Because peak tibial stress increases with gait speed increase and increases in tibial compression during walking may not stem from concurrent increases in vertical ground reaction forces, the physiological loads were estimated to be two times of body weight (70 kgf) to simulate the compressive load on the tibia during static condition [29–31]. Additional 200 N intervention-induced compressive load was uniformly exerted on the tibial osteotomy site in a distracted cortex. The intervention-induced load induced by the stabilizing force on the graft was primarily due to the distraction of the remaining intact cortex, medial collateral ligament, and patellar ligament [23, 24, 32]. According to the results from a previous study, restoration of physiological transfer of a knee load was assumed in the present study, which led to load repartitions of 60% and 40% on the medial and lateral tibial plateaus, respectively (Fig. 2a). The components of the physiological (1,400 N) and surgical loads (200 N) on the four regions of the tibial plateau are shown in Fig. 2b [33].

Three parameters were used to compare the differences in stress, strain, and micromotion with variations in bone formation. First, peak stress (peak von Mises stress, PVMS) was investigated in the cortical bone and lateral hinge area in the FE models, and the stress values were compared to the yield strength to verify stability. The yield strength of the tibial cortical bone (177.2 MPa for axial compression) was obtained from a previous publication [34]. Next, the maximum principal strain in the bones was evaluated. Third, change in gap (△= gap distance before applying load – gap distance after applying load) at the medial edges of the opening gap was evaluated to compare bony stability (Fig. 2c). The present study used 1st principal stress values and the FE analysis was conducted using ABAQUS (version 6.14; Dassault Systems, France).

### Intact model validation

The FE model of normal tibia was validated by comparing it with data from a previous study that experimentally validated an FE model of a human cadaveric tibia [35]. The FE model is validated under axial loading conditions. The minimum and maximum principal strains were investigated. The largest values of the minimum principal strain on the tibia bone and reference model were − 542 and − 569 microstrain, respectively. The largest values of the maximum principal strain on the tibia and reference model were 403 and 426 microstrain, respectively. We considered that the constructed FE model was reliable because the differences were less than 10%. Therefore, the FE model constructed in this study was appropriate for testing by comparing the differences in the largest values of the minimum and maximum principal strains on the tibia between the FE model and previous literature data.

## Results

### Stress distribution on the bone model with plate removal after MOWHTO

**Fig. 3:**
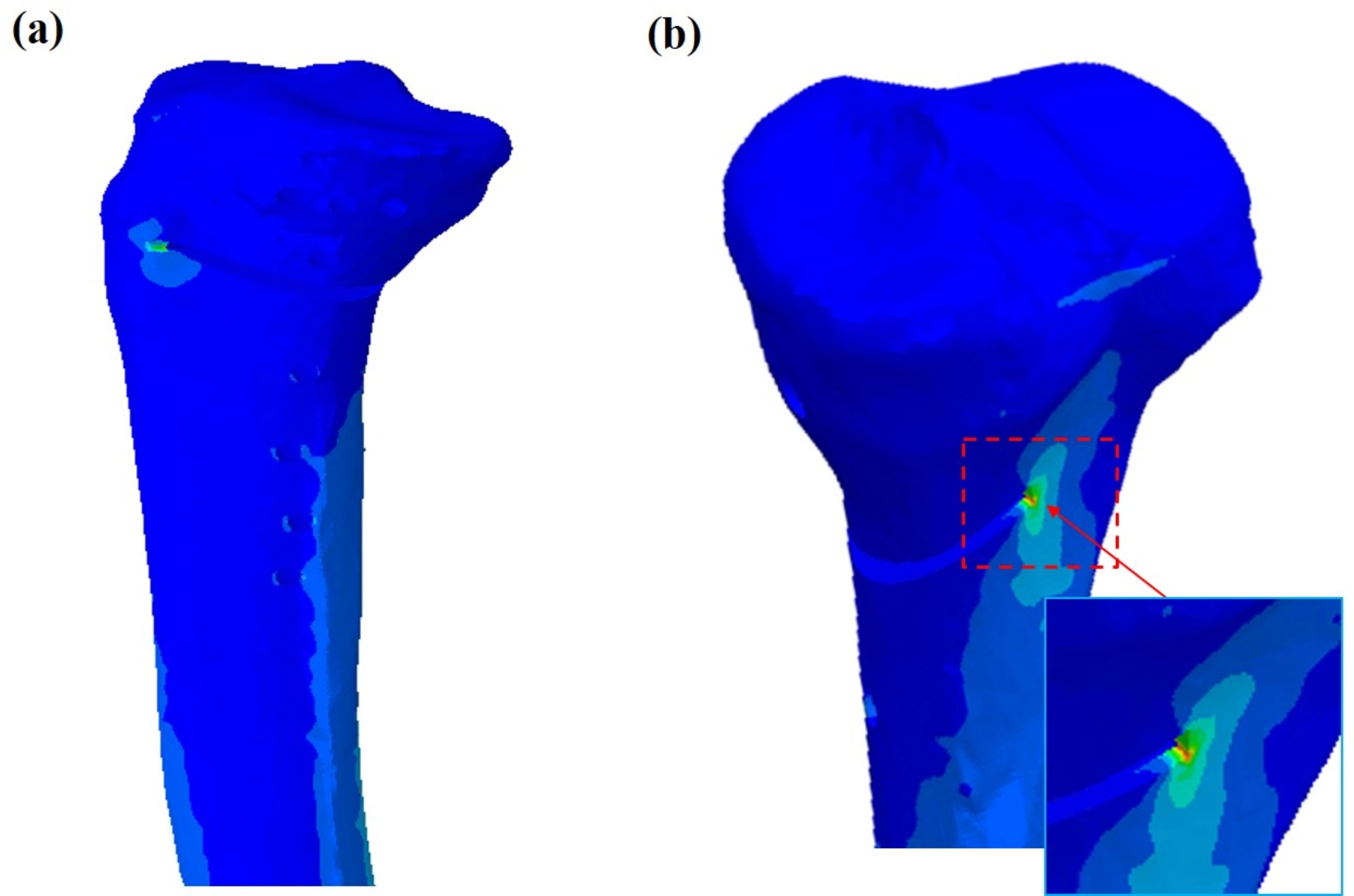
Stress distribution around the proximal tibia with opening gap of 5 mm filled with zone 3 (60%) bone formation after plate removal; medial view (**a**) and posterior view (**b**). The arrow indicates that the peak stress was observed at the posterolateral osteotomy wedge site

Peak von Mises stress was greatest around the posterolateral end of the osteotomy wedge in all the models, and tended to decrease with progressive gap filling (Fig. 3).

**Fig. 4:**
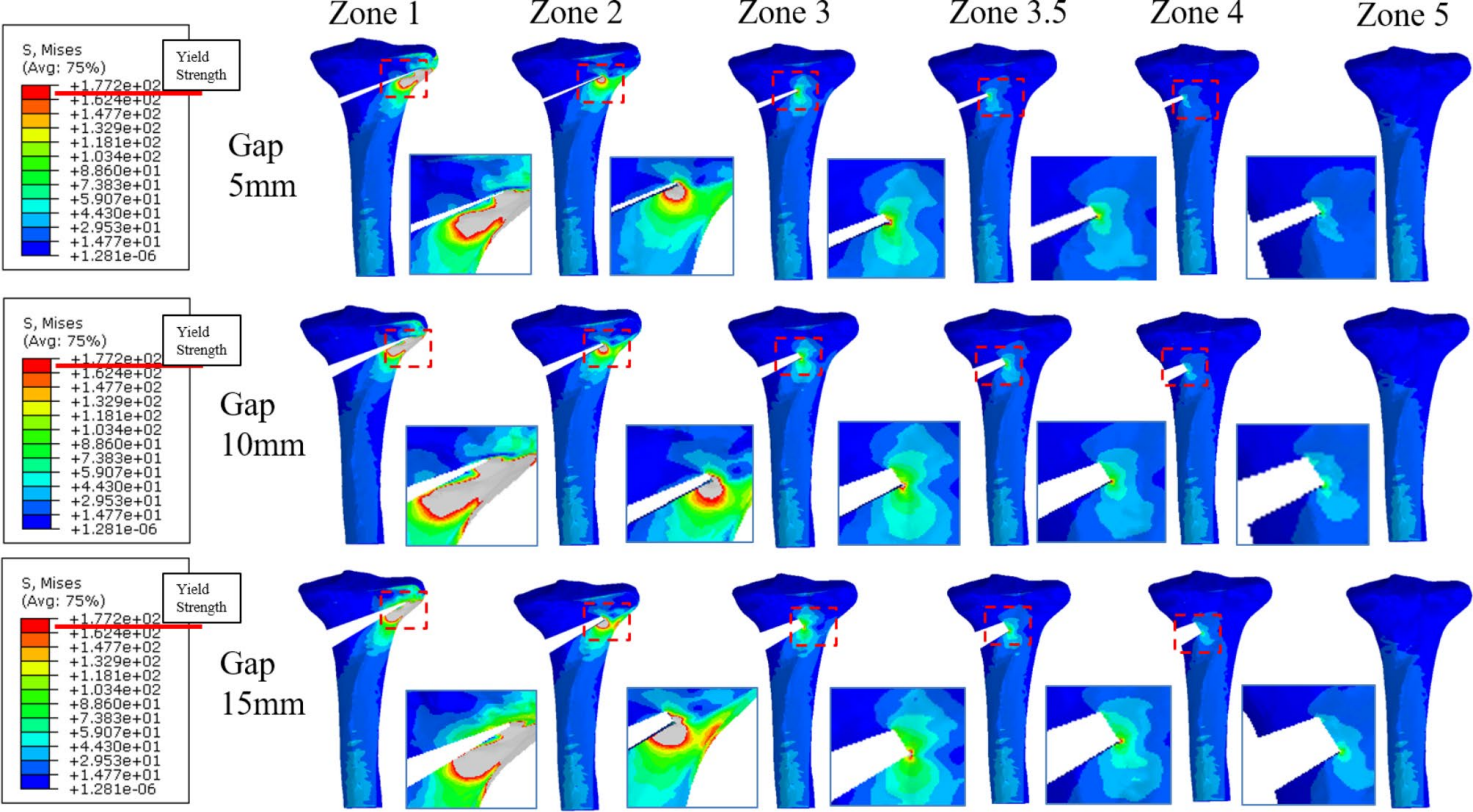
Stress distribution around the proximal tibia with opening gaps of 5 mm (top), 10 mm (middle), and 15 mm (bottom) after plate removal (posterior view). The enlarged image portion represents the point at which the peak stress was observed

In models with osteotomy gaps of 5 and 10 mm, PVMS was greater than yield strength (177.2 MPa) when bone formation reached zone 3 (60%) or less; however, PVMS was lower than yield strength when bone formation reached zone 3.5 (70%) or more (Fig. 4a and b).

In models with 15 mm gaps, PVMS was greater than yield strength when bone formation reached zone 3.5 (70%) or less (Fig. 4c); however, PVMS was lower than yield strength when bone formation reached zone 4 (80%) or more (Table 1).

**Table 1:** Peak von Mises stress (PVMS) at bone models

Opening Gap (mm)	Peak Von Mises Stress (MPa)						
	Zone 1	Zone 2	Zone 2.5	Zone 3	Zone 3.5	Zone 4	Zone 5
Gap 5	4230	1009	769	286	159*	101*	39*
Gap 10	2707	794	455	285	171*	128*	39*
Gap 15	1954	855	589	336	231	127*	39*

* Lower than the yield strength: axial compression 177.2 MPa

### Strain distribution on the bone model with plate removal after MOWHTO

Strain was also greatest around the posterolateral end of the osteotomy wedge in all the models and tended to decrease with gap-filling progression.

**Fig. 5:**
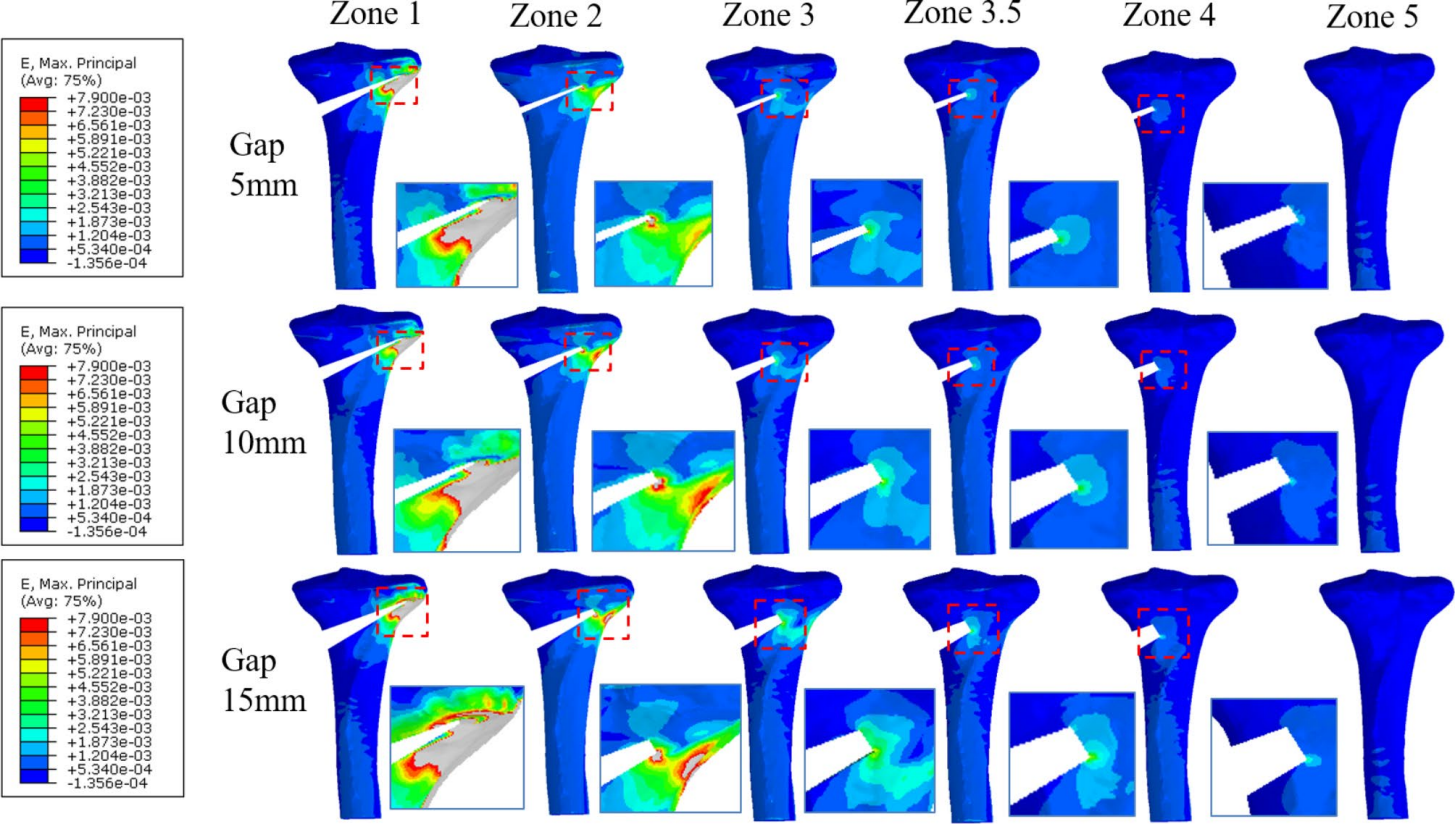
Strain distribution arournd the proximal tibia with opening gaps of 5 mm (top), 10 mm (middle), and 15 mm (bottom) after plate removal (posterior view). The enlarged image portion represents the point at which the maximal strain was observed

Maximal principal strain was greater than 10,000 microstrain when bone formation reached zone 3 (60%) or less in models with osteotomy gaps of 5, 10, and 15 mm; however, it was lower than 6,130 microstrain when bone union reached zone 3.5 (70%) or more in all the models (Fig. 5) (Table 2).

**Table 2:** The maximum principal strain

Opening Gap (mm)	Maximum principal strain ($\:\times\:{10}^{-6}$)						
	Zone 1	Zone 2	Zone 2.5	Zone 3	Zone 3.5	Zone 4	Zone 5
Gap 5	170,000	90,700	44,900	10,100	5920	3470	1580
Gap 10	107,300	76,400	31,400	11,100	5470	3850	1580
Gap 15	96,500	88,000	34,800	10,500	6130	3030	1580

### Micromotion at the edges of the opening gap

As the osteotomy correction gap increased, change in gap at the edges of the opening gap tended to increase. Change in gap at the edges of the osteotomy gap tended to increase from anterior to posterior (aa’< bb’< cc’) (Table 3).

**Table 3:** Change in height at the medial edges of the opening gap

Gap (mm)	Change in the height at medial edges (mm)															
	Zone1	Zone 2			Zone 2.5			Zone 3			Zone 3.5			Zone 4		
	aa’, bb’, cc’	aa’	bb’	cc’	aa’	bb’	cc’	aa’	bb’	cc’	aa’	bb’	cc’	aa’	bb’	cc’
Gap 5	> 5	1.61	1.83	1.86	1.02	1.12	1.14	0.34	0.37	0.37	0.18	0.19	0.19	0.10	0.11	0.11
Gap10	> 10	2.12	2.31	2.38	0.98	1.00	1.01	0.43	0.46	0.47	0.24	0.25	0.26	0.15	0.16	0.17
Gap15	> 15	2.78	2.99	3.05	1.53	1.65	1.69	0.56	0.60	0.61	0.32	0.34	0.35	0.21	0.22	0.22

## Discussion

The most critical finding of the present study is that PVMS was lower than yield strength when bone formation reached zone 3.5 (70%) or more in the 5- and 10-mm gap models, and zone 4 (80%) or more in the 15-mm gap model. However, PVMS was greater than yield strength when bone formation reached zone 3 (60%) or less in the 5- and 10-mm gap models, and zone 3.5 (70%) or less in the 15-mm gap model, which could lead to bone fracture or correction loss after metal plate removal.

The maximum stress and strain in zones 1*–*4 were measured at the posterolateral end of the wedge, which is thought to be due to the loading, was more concentrated in the posterior direction than the anterior. The maximal principal strain was lower than 6,130 microstrain when bone union reached zone 3.5 (70%) or more in all the models; these were lower than 7,900 microstrain, which was previously reported to cause low cycle (< 10,000 cycles) fatigue microcracking [36]. Micromotion at the medial edge of the opening gap tended to increase from anterior to posterior, which was thought to be due to the higher load on the posterior part than the anterior part. As the osteotomy correction gap increased, change in gap at the edges of the opening gap tended to increase; greater micromotion may lead to potentially unstable proximal tibial structure, but the risk of correction loss was assessed based on the maximum stress and strain results.

When considering metal plate removal to relieve discomfort and obtain clinical benefits, no clear agreement exists on the degree of bone formation that allows hardware removal without correction loss after MOWHTO. Brinkman et al. [17] reported that complete union was achieved in 90% 1 year after the surgery and recommended that the metal plate should not be removed before 1.5 years after MOWHTO. However, there is no consensus that plate removal is possible only when complete union is achieved, and many surgeons attempt to remove the plate before complete union. Goshima et al. [37] reported that metal plate removal without correction loss is possible if gap filling arrives at zone 2 (25–50%) on simple AP X-ray radiographs; however, in another follow-up study, they experienced loss of correction in 6 (5.9%) of 101 patients who underwent plate removal according to these criteria [19].

In a follow-up study, Goshima et al. [19] reported in their follow-up study that posterior cortical-bone union rate was the only predictor of correction loss. They suggested using receiver operating characteristic curve analysis cut-off value that posterior cortex bone union rate of < 43.3% caused an increase in posterior tibial slope and decrease in medial proximal tibial angle [19]. They finally concluded that when the posterior cortical bone union reached the gap center (50%), the plate could be removed without loss of correction. However, statistical analysis using data from the 6(5.9%) of 101 correction loss cases seems to have limitations in representativeness. In addition, they considered the correction loss only from a coronal plane point of view, not including a sagittal plane aspect such as posterior tibial slope change; therefore, biomechanical analysis using an FE model to determine the degree of bone formation extent where the plate can be removed without correction loss considering both coronal and sagittal plane aspects seems to be meaningful. According to the present study, PVMS was lower than yield strength when bone formation reached zone 3.5 (70%) or more in 5- and 10-mm osteotomy gap models, and zone 4 (80%) or more in a 15-mm gap model. Our study results suggest that it would be safer to perform plate removal after obtaining sufficient bone formation rather than performing it near the osteotomy gap center (50%) to avoid correction loss despite continued patient discomfort.

The hypothesis of this study was to question the previous clinical study report that > 50% bone formation was safe from correction loss, and to establish that there may be a safe zone even in < 50% bone formation on anterior-posterior (AP) radiographs. However, the results of our study did not support the null hypothesis of the present study, suggesting that more bone formation than the degree of bone formation suggested in the hypothesis was necessary to achieve mechanical stability of the proximal tibia after metal plate removal. The difference between the null hypothesis and the present study results may be due to the different perspective regarding the term “correction loss”. The previous clinical report by Goshima et al. considered correction loss as a varus change of 2 degrees or more in the coronal plane lower extremity mechanical axis over 1 year. In our study, when < 70% bone formation was progressed, peak stress measured at the posterolateral part of the osteotomy wedge was higher than yield stress, which suggested a possible risk of bony fracture and potential correction loss not only in coronal plane aspect, but also in sagittal plane perspective. Clinical research should be followed for matching the present FE model results with clinical situations.

The present study has several limitations. First, simple assumptions in material properties (linear homogenous isotropic material properties), uniform bone healing, and the simplified geometry of the gap were applied in this study. Second, this study was conducted under static conditions because dynamic knee joint motion is too complicated in terms of computing resources and time. In future studies, a more suitable representation of the mobile joint and study of the models under cyclic loading should be conducted. Third, only the tibia was simulated in this study. Expanding the scope of the cartilage may be required to investigate the pressing biomechanics of MOWHTO. Forth, our results cannot be generalized to patients with osteoporosis. Fifth, the present study used a single anatomical model (a 62-year-old Asian woman), which limits generalizability. Future studies should include multiple anatomical models to enhance applicability to diverse patient populations. Sixth, because the model constructed in this research was based on living subject, our research model could not be validated using mechanical test of full cadaver model.

However, to the best of our knowledge, this is the first FE analysis to demonstrate the appropriate timing of metal plate removal without correction loss after MOWHTO. The results of the biomechanical mechanics based on this study will contribute to the body of decision-making regarding the timing of plate removal after MOWHTO.

## Conclusions

The present study demonstrated that plate removal seems to be possible without correction loss after MOWHTO when bone formation reached zone 3.5 (> 70%) or more during 5- and 10-mm osteotomy gap corrections, and zone 4 (> 80%) or more during 15-mm gap correction. Our study results suggest that it would be safer to perform plate removal after obtaining sufficient bone formation rather than performing it near the osteotomy gap center (50%) to avoid correction loss considering both coronal and sagittal plane aspects despite continued patient discomfort.

## Data Availability

No datasets were generated or analysed during the current study.

## Abbreviations

MOWHTO: Medial opening-wedge high tibial osteotomy
FE: Finite element
AP: Anterior-posterior
CT: Computed tomography
CAD: Computer-aided design
PVMS: peak von Mises stress
